# Supplementary material for: Origin and differentiation trajectories of fibroblastic reticular cells in the splenic white pulp
Source: Nat Commun. 2019 Apr 15;10:1739. doi: 10.1038/s41467-019-09728-3 (PMC6465367; doi:10.1038/s41467-019-09728-3)
Supplement: Supplementary file 1 — Supplementary Information [file 41467_2019_9728_MOESM1_ESM.pdf]

Supplementary information

Origin and differentiation trajectories of fibroblastic reticular cells in the splenic white pulp

Cheng et al

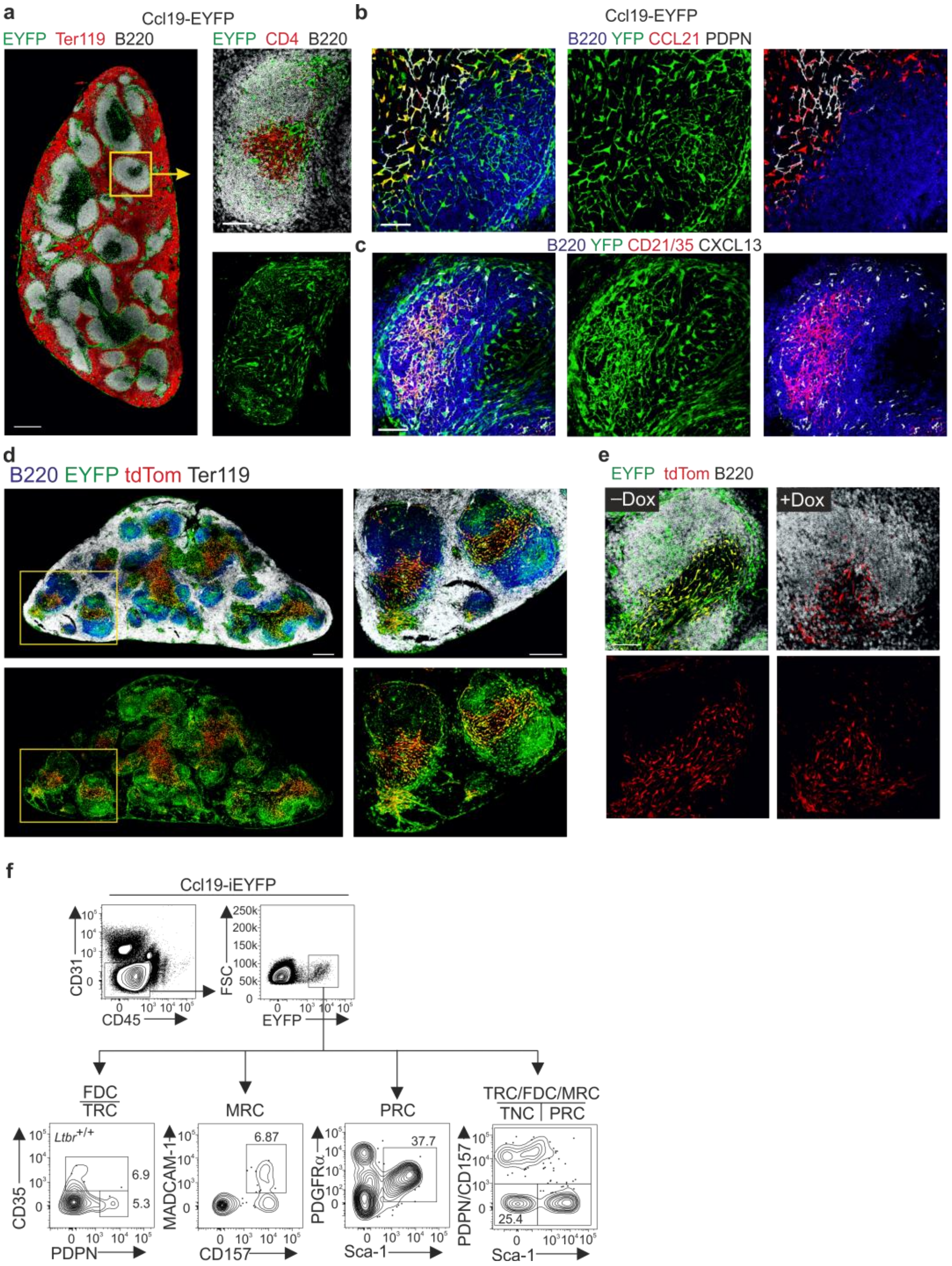

Supplementary Figure 1. Genetic targeting of white pulp fibroblastic stromal cells in Ccl19-EYFP mice. (a) Confocal microscopy of spleen cross-sections from adult Ccl19-EYFP mice stained with the indicated antibodies. Box indicates magnified white pulp area in right panels (scale bars = 500 µm/50 µm) (b-c) $EYFP^{+}$ reticular cell subsets in splenic white pulp areas of adult Ccl19-EYFP mice analyzed by confocal microscopy after staining with the indicated antibodies, scale bar = 20 µm. (d) Confocal microscopy of EYFP and tdTomato (tdTom) expression in adult Ccl19-iEYFP spleens stained with indicated antibodies (scale bars = 300 µm/200 µm). (e) Confocal microscopy analysis of EYFP and tdTomato expression in adult Ccl19-iEYFP spleens. Mice were kept untreated (-Dox) or treated lifelong with doxycycline (+Dox). Representative data set from 2 independent experiments (n = 4 mice), scale bar = 50 µm. (f) Representative flow cytometric analysis of splenic fibroblastic reticular cells (FRC) isolated from Ccl19-EYFP mice. Gating strategy was used for analysis of respective FRC subsets in the main Figures 1 and 2.

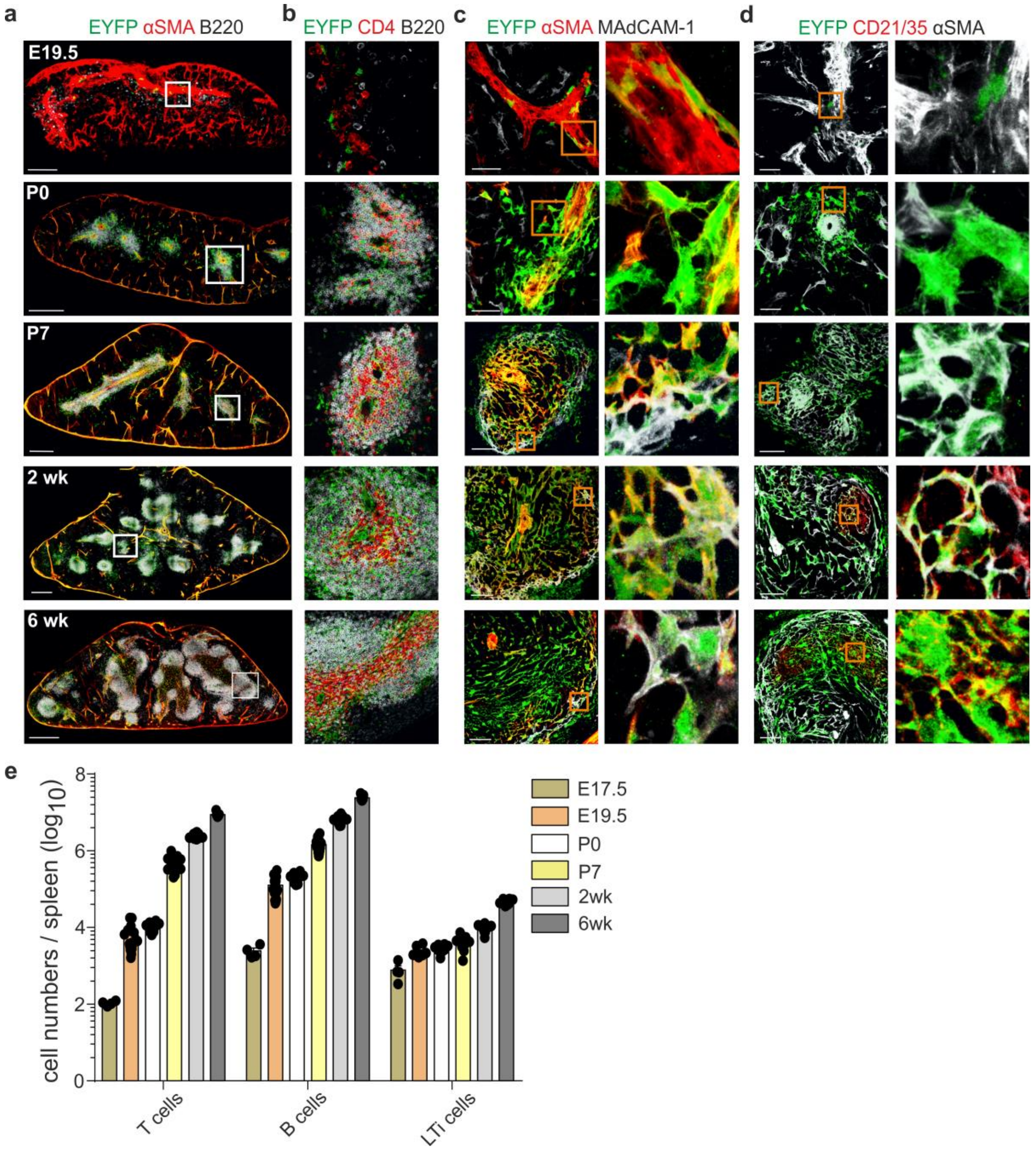

Supplementary Figure 2. Splenic white pulp development in Ccl19-iEYFP mice. (a-b) Cross-sections of spleens from Ccl19-iEYFP mice at the indicated age, stained with antibodies directed to the indicated markers and analyzed by confocal microscopy. Boxed area indicates representative area shown at higher magnification in (b) (scale bars = 50 µm for E19.5, 100 µm for P0 and P7, 200 µm for 2wk and 500 µm for 6 wk). (c and d) High resolution confocal microscopy analysis of $EYFP^{+}$ reticular cells using the indicated antibodies; boxed areas show region of higher magnification in right images (scale bars = 20 µm for E19.5 and P0, 30 µm for P7, 2 wk and 6 wk). (e) Hematopoietic cell populations in spleens at different stages revealed by flow cytometry (n = 4-16 per group from 2-3 independent experiments). Statistical analysis was performed using Mann-Whitney test. Microscopy data are representative for 2 or more independent experiments (n ≥ 3 per group). Source data are provided as Source Data file.

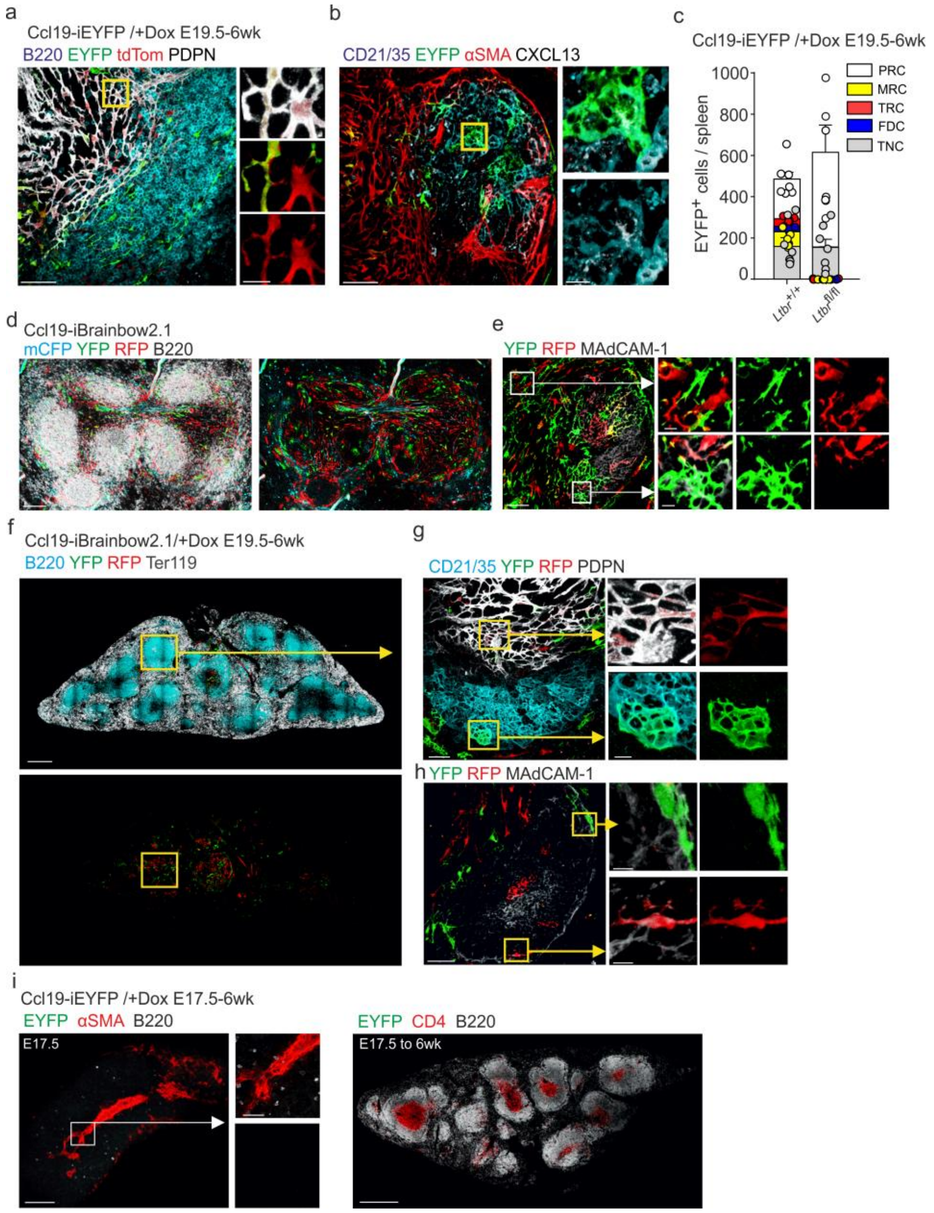

Supplementary Figure 3. Cell fate-mapping of FRC progenitors in Ccl19-iEYFP and Ccl19-iBrainbow2.1 mice. (a-b) Confocal microscopic analysis of chemokine expression in E19.5 to 6 weeks fate-mapped spleens stained with indicated markers (scale bars = 50 μm) and boxed area indicates representative area shown at higher magnification. (scale bars = 10 μm). (c) Absolute numbers FRC subsets recovered from E19.5 to 6 weeks fate-mapped spleens of Ccl19-iEYFP and Ccl19-iEYFP *Ltbr*$^{fl/fl}$ mice (n=7). (d-e) Confocal microscopic analysis of CFP, YFP and RFP in Ccl19-iBrainbow2.1 spleens costained with anti-B220 (scale bars = 50 μm) or anti-MAdCAM-1 (scale bars = 50 μm). Boxed area in (e) indicates representative area shown at higher magnification (scale bars = 10 μm). (f-h) Confocal microscopy analysis of YFP and RFP expression in E19.5 to 6 weeks fate-mapped spleens from Ccl19-iBrainbow2.1 mice stained with the indicated markers (scale bar in g = 300μm, h and i = 50μm) and boxed area indicates representative area shown at higher magnification (scale bar in h and i = 10μm). (n =5 from 2 independent experiments) (i) Spleens were harvested from Ccl19-iEYFP embryos at E17.5 and from 6 weeks old Ccl19-iEYFP mice treated with doxycycline (Dox) from E17.5 onwards. Sections were stained with antibodies against EYFP, αSMA and B220 and analyzed by confocal microscopy. Boxed areas indicate region of higher magnification shown in right images (scale bars in left image = 100/20 μm, scale bar in right image = 500 μm). Microscopy data are representative for 2 independent experiments (n ≥ 2 per group). Source data are provided as Source Data file.

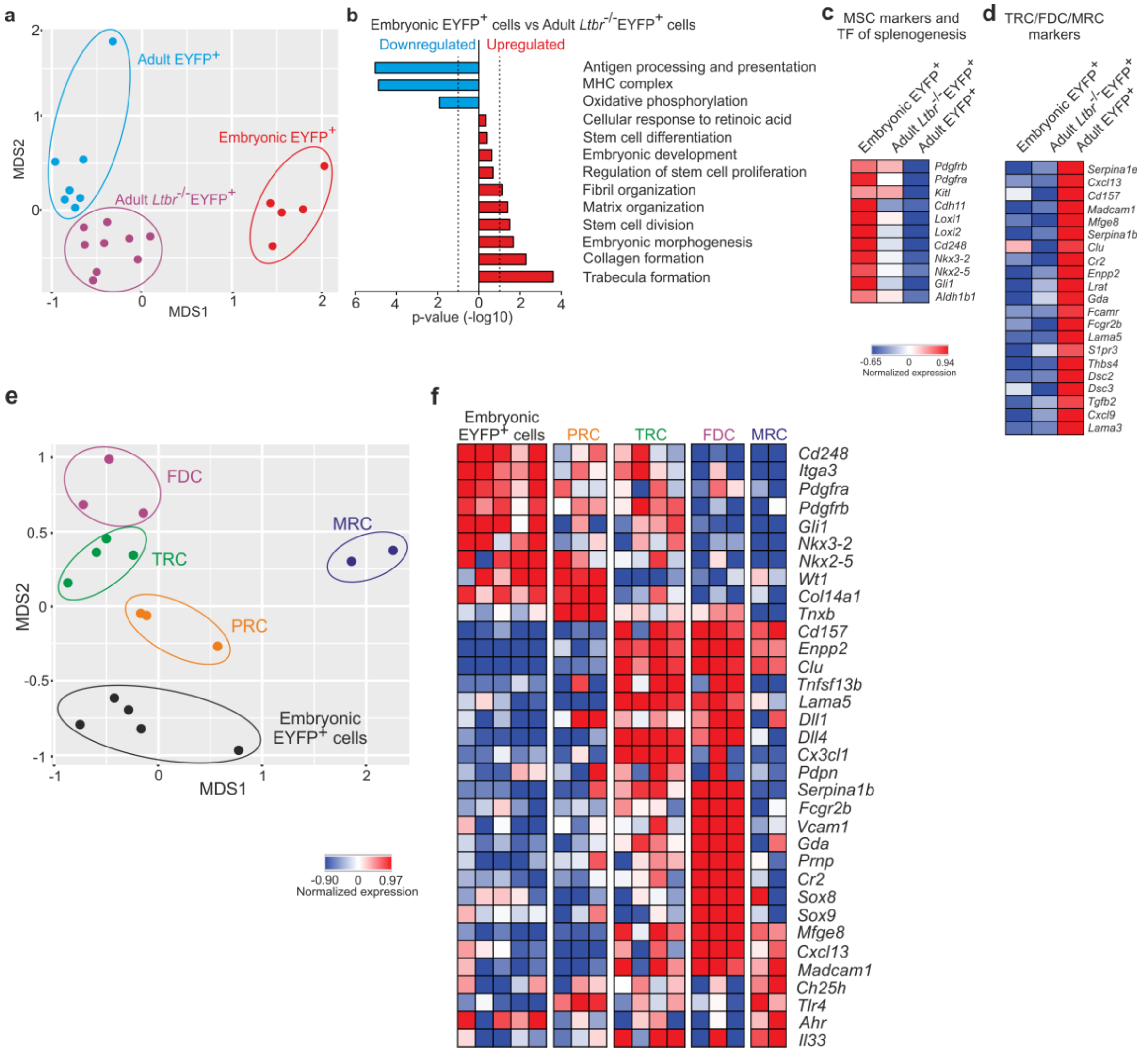

Supplementary Figure 4. Transcriptomic analysis of embryonic and adult reticular cells. (a) Multidimensional scaling (MDS) plot based on differentially expressed genes (adjusted *p*-value < 0.05 in any pairwise comparison) from RNA-seq dataset of EYFP$^+$ cells from spleens of embryonic and adult Ccl19-iEYFP, and Ccl19-iEYFP *Ltbr*$^{fl/fl}$ mice (n = 5-9 mice per group from 3 independent experiments). (b) Gene set analysis in embryonic EYFP$^+$ cells and adult EYFP$^+$ cells lacking *Ltbr* expression. Data represent FDR-adjusted *p*-values (-$\log_{10}$ scale) for enrichment in indicated gene sets. Statistical analysis was performed using a competitive gene set test accounting for inter-gene correlation. (c and d) Genes related to LTβR-dependent mesenchymal stem cell (MSC) markers and transcription factors (TF) involved in splenic organogenesis (c) and TRC/FDC/MRC markers (d). (e) Multidimensional scaling (MDS) plot based on differentially expressed genes (adjusted *p*-value < 0.05 in any pairwise comparison) from RNA-seq dataset of EYFP$^+$ cells sorted from spleens of embryonic Ccl19-iEYFP mice and PRC, MRC, TRC and FDC subsets from adult Ccl19-iEYFP mice. (f) Heatmap of genes related to embryonic EYFP$^+$ cells, PRC, TRC, FDC and MRC populations. Data represent logCPM values which have been row-mean centered, row-normalized and averaged for all samples within each group.

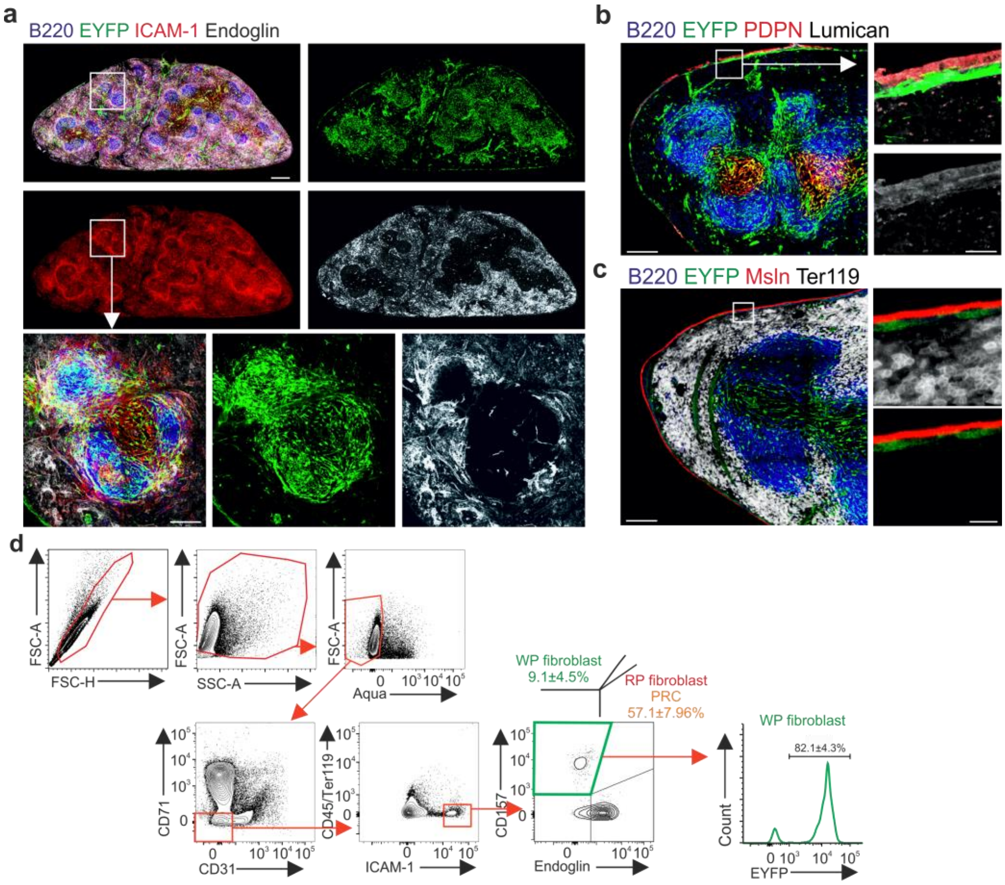

Supplementary Figure 5. Identification of splenic fibroblastic stromal cell subsets. (a-c) Cross-sections of spleens from Ccl19-iEYFP mice stained with antibodies directed to the indicated markers and analyzed by confocal microscopy (scale bar in a = 300 µm, b and c = 100 µm). Boxed area indicates representative area shown at higher magnification (scale bar in a = 100 µm, b = 30 µm, c = 15 µm). (n=4 from 2 independent experiments) (d) Representative flow cytometric analysis of fibroblastic stromal cells from Ccl19-iEYFP spleens without doxycycline treatment and analyzed for the indicated markers. Histogram in the right panel indicates the EYFP expression in ICAM-1^{+} CD157^{+} Endoglin^{-} white pulp fibroblasts (n = 6 mice per group from 3 independent experiments, mean ± SEM). Source data are provided as Source Data file.

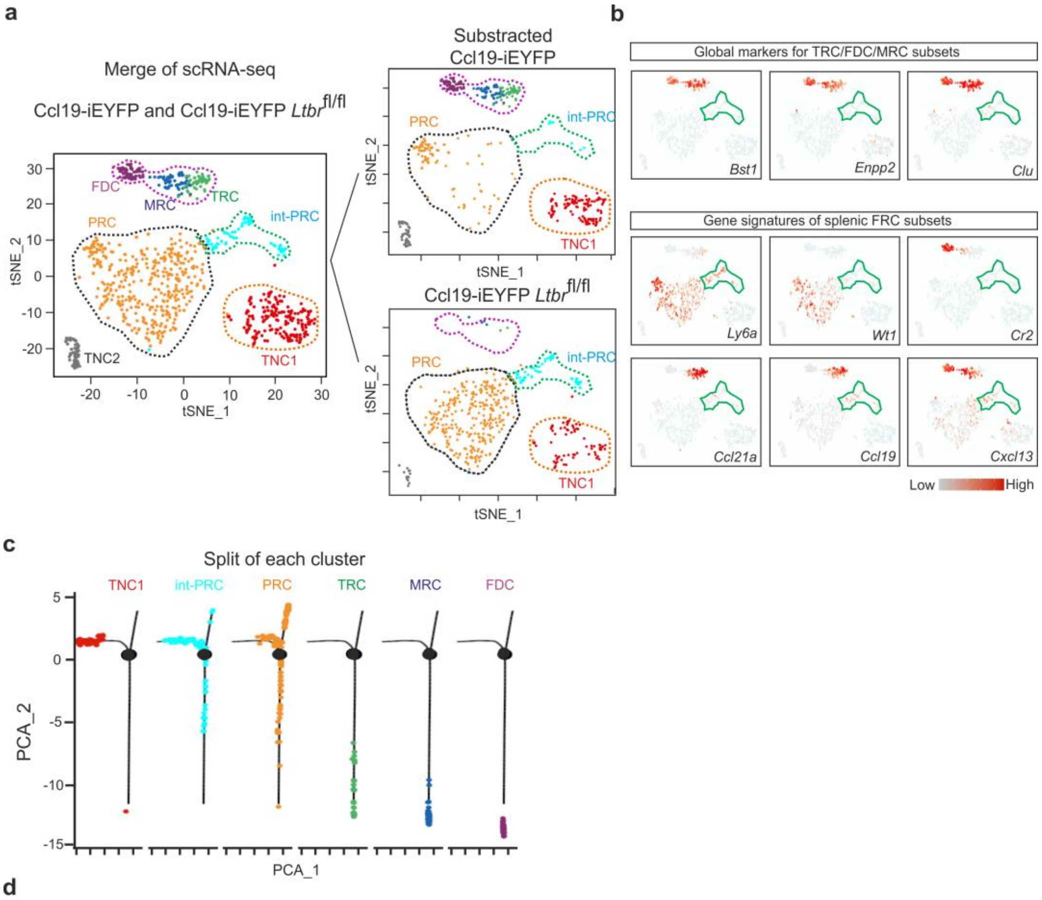

d

Subsets	Selected marker genes	Enriched pathway	Biological function
TRC	*Ccl21a, Ccl19, Clu, Il33, Dcn, Bst1,C3,Thbs4,Il7, Cd74, Enpp2, Dsc2, Pdpn*	Chemokine mediated signaling<br>Humoral immune response<br>IFN response	Lymphocyte migration<br>Monocyte migration
MRC	*Cxcl13, Mfge8, Ccl21a, Trf, Madcam1, C3, Ctss, Cfb, Cxcl9, Bst1, Enpp2*	Humoral immune response<br>Antigen presentation<br>Complement activation	Antigen presentation<br>Complement response
FDC	*Cxcl13, Mfge8, Cr2, Pthlh, Madcam1, Il4ra, Klf6, Sox9, Fcgr2b, Postn, Fnbp1*	Antigen presentation<br>TNFR signaling	Antigen presentation<br>Lymphocyte activation
PRC	*Ly6a, Cyp4b1, Tnx10b, Cxcl12, Coch, Tlx1, Pdgfra, Pdgfrb, Wt1, Col14a1*	Extracellular matrix production<br>Mesenchymal stem cell<br>IFN response	ECM organization<br>MSC function
TNC1<br>Mural cells	*Acta2, Myh11, Rgs5, Tagln, Mcam, Cnn1, Esam, Cspg4, Notch3, Jag1, Mef2c*	Cell contractility<br>Oxidative phosphorylation	Vascular wall stability
TNC2	*Mki67, Mcm7, Cdk6, Cks2, Car1, Car2, Sox4,*	Cell cycle<br>Translation initiation	Maintenance of<br>FRC and/ or mural cells

Supplementary Figure 6. Transcriptomic analysis and differentiation trajectories of LTβR-proficient and -deficient reticular cell subsets. (a) Merged t-distributed stochastic neighbor embedding (t-SNE) cell map of sorted splenic EYFP$^+$ cells from adult Ccl19-iEYFP and Ccl19-iEYFP *Ltbr*$^{fl/fl}$ spleen highlight the distinct reticular subsets (left panel). The subtraction of individual dataset from Ccl19-iEYFP and Ccl19-iEYFP *Ltbr*$^{fl/fl}$ samples are shown in the right panel. (b) Gene signatures of distinct splenic reticular cell subsets acquired from scRNA-seq analysis. The density of the red color represents the expression level of the genes. Green demarcation highlights the int-PRC population in the t-SNE. (c) The split of each cell clusters situated in the trajectory routes constructed via Monocle analysis. (d) Summary of marker genes and enriched pathway in distinct splenic reticular cell subsets based on the scRNA-seq readout. Enriched pathways are characterized based on the gene set enrichment assay (GSEA). Data represent FDR-adjusted *p*-values ($-\log_{10}$ scale < 1) for enrichment in indicated gene sets.
