# Supplementary material for: Origin and differentiation trajectories of fibroblastic reticular cells in the splenic white pulp
Source: Nat Commun. 2019 Apr 15;10:1739. doi: 10.1038/s41467-019-09728-3 (PMC6465367; doi:10.1038/s41467-019-09728-3)
Supplement: Supplementary file 3 — Reporting Summary [file 41467_2019_9728_MOESM3_ESM.pdf]

nature research

Corresponding author(s): Burkhard Ludewig

Last updated by author(s): 19.March.2019

Reporting Summary

Nature Research wishes to improve the reproducibility of the work that we publish. This form provides structure for consistency and transparency in reporting. For further information on Nature Research policies, see Authors & Referees and the Editorial Policy Checklist.

Statistics

For all statistical analyses, confirm that the following items are present in the figure legend, table legend, main text, or Methods section.

n/a	Confirmed	
☐	☒	The exact sample size (n) for each experimental group/condition, given as a discrete number and unit of measurement
☐	☒	A statement on whether measurements were taken from distinct samples or whether the same sample was measured repeatedly
☐	☒	The statistical test(s) used AND whether they are one- or two-sided<br>*Only common tests should be described solely by name; describe more complex techniques in the Methods section.*
☒	☐	A description of all covariates tested
☐	☒	A description of any assumptions or corrections, such as tests of normality and adjustment for multiple comparisons
☐	☒	A full description of the statistical parameters including central tendency (e.g. means) or other basic estimates (e.g. regression coefficient) AND variation (e.g. standard deviation) or associated estimates of uncertainty (e.g. confidence intervals)
☐	☒	For null hypothesis testing, the test statistic (e.g. F, t, r) with confidence intervals, effect sizes, degrees of freedom and P value noted<br>*Give P values as exact values whenever suitable.*
☒	☐	For Bayesian analysis, information on the choice of priors and Markov chain Monte Carlo settings
☐	☒	For hierarchical and complex designs, identification of the appropriate level for tests and full reporting of outcomes
☒	☐	Estimates of effect sizes (e.g. Cohen's d, Pearson's r), indicating how they were calculated

Our web collection on statistics for biologists contains articles on many of the points above.

Software and code

Policy information about availability of computer code

Data collection	n.a.
Data analysis	FlowJo version 7 and 10<br>GraphPad Prism 7.01<br>Imaris V8.0.2<br>R v3.4.0

For manuscripts utilizing custom algorithms or software that are central to the research but not yet described in published literature, software must be made available to editors/reviewers. We strongly encourage code deposition in a community repository (e.g. GitHub). See the Nature Research guidelines for submitting code & software for further information.

Data

Policy information about availability of data

All manuscripts must include a data availability statement. This statement should provide the following information, where applicable:

- Accession codes, unique identifiers, or web links for publicly available datasets
- A list of figures that have associated raw data
- A description of any restrictions on data availability

RNAseq and scRNA-seq data are deposited in the arrayexpress database (www.ebi.ac.uk/arrayexpress) under accession numbers E-MTAB-7703 [https://www.ebi.ac.uk/arrayexpress/experiments/E-MTAB-7703/], E-MTAB-7097 [https://www.ebi.ac.uk/arrayexpress/experiments/E-MTAB-7097/] and E-MTAB-7094 [https://www.ebi.ac.uk/arrayexpress/experiments/E-MTAB-7094/]. Data will be accessible after the acceptance of the paper.

Field-specific reporting

Please select the one below that is the best fit for your research. If you are not sure, read the appropriate sections before making your selection.

☒ Life sciences ☐ Behavioural & social sciences ☐ Ecological, evolutionary & environmental sciences

For a reference copy of the document with all sections, see nature.com/documents/nr-reporting-summary-flat.pdf

Life sciences study design

All studies must disclose on these points even when the disclosure is negative.

Sample size	All flow cytometric analyses were routinely performed at sample sizes >5 mice per group, which are sufficient to generate statistically significant results.
Data exclusions	No data were excluded from the study.
Replication	All analyses have been replicated in at least 2 independent experiments.
Randomization	All mice in this study were typed for transgene positivity, while negatively typed littermates were used as control.
Blinding	No investigator blinding was used in this study.

Reporting for specific materials, systems and methods

We require information from authors about some types of materials, experimental systems and methods used in many studies. Here, indicate whether each material, system or method listed is relevant to your study. If you are not sure if a list item applies to your research, read the appropriate section before selecting a response.

Materials & experimental systems

n/a	Involved in the study	
☐	☒	Antibodies
☒	☐	Eukaryotic cell lines
☒	☐	Palaeontology
☐	☒	Animals and other organisms
☒	☐	Human research participants
☒	☐	Clinical data

Methods

n/a	Involved in the study	
☒	☐	ChIP-seq
☐	☒	Flow cytometry
☒	☐	MRI-based neuroimaging

Antibodies

Antibodies used	Clone eBio1D3 anti-CD19 PE-Cy7 1:200 Thermo Fischer Scientific; Clone MEC13.3 anti-CD31 Alexa fluor-647 1:200 Biolegend; Clone MEC13.3 anti-CD31 PerCP 1:200 Biolegend ; Clone 145-2C11 anti-CD3e FITC 1:200 Biolegend; Clone 30F-11 anti-CD45 APC-Cy7 1:200 Biolegend; Clone RA3-6B2 anti-B220 Alexa fluor-647 1:500 Biolegend; Clone 8.1.1 anti-PDPN PE 1:200 Biolegend; Clone TER-119 anti-Ter-119/Erytroid APC-Cy7 1:100 Biolegend; Clone TER-119 anti-Ter-119/Erytroid Biotin 1:500 Biolegend; Clone BP-3 anti-CD157 APC 1:200 Biolegend; Clone 429 anti-CD106 BV711 1:200 BD Bioscience;Clone HMB1-1 anti-CD29 APC-Cy7 1:200 Biolegend; Clone IM7 anti-CD44 APC 1:200 Biolegend; Clone BP-3 anti-CD157 PE 1:200 Biolegend; Clone APB5 anti-CD140a Biotin 1:100 BioLegend; Clone B2D anti-RORrt PerCP 1:100 Thermo Fischer Scientific; Clone 8C12 anti-CD35 Biotin 1:100 BD Bioscience; Clone MECA-367 anti-MAdCAM-1 Biotin 1:100 BD Bioscience; Clone 7G6 anti-CD21/35 Biotin 1:100 BD Bioscience; Clone D7 anti-Sca-1 PerCP 1:100 BioLegend;Clone RA3-6B2 Anti-CD45R (B220) Alexa fluor-647 1:200 Biolegend; Streptavidin-conjugated PE-Cy7 1:1000 BioLegend; Clone RM4-5 anti-CD4 Biotin 1:500 BioLegend;Clone HMB1-1 anti-CD29 Biotin 1:100 Biolegend; Clone SolA15 anti-Ki-67 Biotin 1:100 Thermo Fischer Scientific; Clone: MJ7/18 anti-Endoglin BV785 BD Bioscience 1:200; Clone: MJ7/18 anti-Endoglin Biotin BD Bioscience 1:100; Clone 1A4 anti-αSMA Cy3 1:500 Sigma; Clone JL-8 anti-EYFP 1:500 Takara; Living Colors DsRed Polyclonal Antibody anti-tdTomato 1:500 Takara; Polyclonal Goat IgG anti-CCL21 Biotin 1:100 R&D Systems; Polyclonal Goat IgG anti-CXCL13 Biotin 1:100 R&D Systems; Purified Goat anti-rabbit-IgG Dylight-488 1:500 Jackson Immunotools ; Purified Goat anti-rat-IgG Dylight549 1:500 Jackson Immunotools ; Purified Goat anti-syrian hamster-IgG Dylight549 1:500 Jackson Immunotools ; Purified Streptavidin conjugated Cy3 1:500 Jackson Immunotools ;Purified Streptavidin conjugated Dylight-649 1:500 Jackson Immunotools; Monoclonal Rabbit IgG anti-Calponin-1 1:100 abcam; .
Validation	Antibodies were chosen based on the validation statements for species (mouse) and application (flow cytometry or confocal microscopy) on the manufacturer's website.

Animals and other organisms

Policy information about studies involving animals; ARRIVE guidelines recommended for reporting animal research

Laboratory animals	All mouse strains were on a C57BL/6NCrl genetic background and maintained in individually ventilated cages under specific pathogen free conditions. The BAC-transgenic C57BL/6N-Tg(Ccl19-tTA)688BIAT (Ccl19-tTA) mouse was generated using homologous recombination-mediated transgenesis 58 using a tetracycline transactivator-based single-cassette system for spatiotemporal gene regulation 59. The C57BL/6N-Tg(Ccl19-Cre)489Biat (Ccl19-Cre) (Chai et al., 2013) and the Ltbrtm1.1Thhe (LTβRfl/fl) (Wimmer et al., 2012) strains were described previously. B6.129X1-Gt(ROSA)26Sortm1(EYFP)Cos/J (R26R-EYFP) and B6.129X1-Gt(ROSA)26Sortm1(CAG-Brainbow2.1)Cle (Brainbow2.1) (Livet et al., 2007) mice were purchased from The Jackson Laboratories and the LC1 strain (Schonig et al., 2002) was kindly provided by Dr. Fendler, Max Delbrück Center of Molecular Medicine, Berlin, Germany. Fate-mapping experiments were performed via treating the pregnant dam with 1 mg doxycycline (Sigma Aldrich) intraperitoneally and maintenance of doxycycline in the drinking water (1 mg/ml). Experiments were performed in accordance with Swiss federal and cantonal guidelines (Tierschutzgesetz) under the permission numbers SG13/15, SG16/07 and SG05/17 granted by the Veterinary Office of the Canton of St. Gallen.
Wild animals	n.a.
Field-collected samples	n.a.
Ethics oversight	Experiments were performed in accordance with Swiss federal and cantonal guidelines (Tierschutzgesetz) under the permission numbers SG13/15, SG16/07 and SG05/17 granted by the Veterinary Office of the Canton of St. Gallen.

Note that full information on the approval of the study protocol must also be provided in the manuscript.

Flow Cytometry

Plots

Confirm that:

- [ ] The axis labels state the marker and fluorochrome used (e.g. CD4-FITC).
- [x] The axis scales are clearly visible. Include numbers along axes only for bottom left plot of group (a 'group' is an analysis of identical markers).
- [x] All plots are contour plots with outliers or pseudocolor plots.
- [x] A numerical value for number of cells or percentage (with statistics) is provided.

Methodology

Sample preparation	Spleens were harvested and were perfused with RPMI 1640 medium containing 2% FCS, 20 mM Hepes (all from Lonza), 1 mg/ml Collagenase D (Sigma) and 25 µg/ml DNaseI (Applichem)with 22G a syringe. Samples were teared into smaller pieces and incubated at 37°C for 30 minutes, with resuspension and collection of supernatant every 15 min to PBS containing 1% FCS and 10 mM EDTA (MACS buffer). To enrich fibroblastic stromal cells, hematopoietic and erythrocytes were depleted by incubating the cell suspension with MACS anti-CD45 and anti-TER119 microbeads (Miltenyi Biotec) and passing them through a MACS LS column (Miltenyi Biotec). Unbound single cell suspensions were incubated for 20 minutes at 4°C in PBS containing 1% FCS and 10 mM EDTA with the corresponding labelled antibodies. Cells were acquired with a FACS Canto (BD Biosciences) or a BD LSR Fortessa (BD Biosciences) and analyzed using FlowJo software (Treestar Inc.).
Instrument	FACS Canto (BD Biosciences)<br>BD LSR Fortessa (BD Biosciences)
Software	FlowJo software 7 and 10 (Treestar Inc.).
Cell population abundance	More than 85% purity of sorted EYFP+ cells from Ccl19-iEYFP and Ccl19-iEYFP Ltbrfl/fl spleens are used for total RNA-seq analysis related to (Supplementary figure 4a-d). Similarly, more than 90% purity of sorted recticular cell subsets stained with canonical cell markers are employed for population-based RNA-seq (related to Supplementary figure 4e-f).
Gating strategy	Doublets were excluded using forward light-scatter gating followed by gating on lymphocytes based on FSC-SSC. Stromal cells were gated from the the CD45-TER119- cell population; for the hematopoietic cell analysis, the CD45+ population was gated.

- [x] Tick this box to confirm that a figure exemplifying the gating strategy is provided in the Supplementary Information.
