# Supplementary material for: Origin and differentiation trajectories of fibroblastic reticular cells in the splenic white pulp
Source: Nat Commun. 2019 Apr 15;10:1739. doi: 10.1038/s41467-019-09728-3 (PMC6465367; doi:10.1038/s41467-019-09728-3)
Supplement: Supplementary file 4 — Description of Additional Supplementary Files [file 41467_2019_9728_MOESM4_ESM.pdf]

Description of Additional Supplementary Files

Supplementary Movie 1. Embryonic lymphoid tissue organizer cells surround the splenic artery. Splenic anlage has been harvested from Ccl19-iEYFP embryos (E19.5), stained with antibodies directed to EYFP and αSMA and imaged in whole mount by confocal microscopy.
